# A Consistent Round-Up Strategy Based on PPO Path Optimization for the Leader–Follower Tracking Problem

**DOI:** 10.3390/s23218814

**Published:** 2023-10-30

**Authors:** Xiao Wang, Zhaohui Yang, Xueqian Bai, Mingjiang Ji, Hao Li, Dechao Ran

**Affiliations:** 1 College of Information Science and Technology, Beijing University of Chemical Technology, Beijing 100029, China; w_xiao@buct.edu.cn （X.W.); 2020040414@buct.edu.cn (Z.Y.); 2020040319@buct.edu.cn (X.B.); 2 National Innovation Institute of Defense Technology, Academy of Military Sciences, Beijing 100071, China; jimingjiangnet@126.com; 3 The Second Academy of CASIC, Beijing 100854, China; lihao_guazi@163.com

**Keywords:** reinforcement learning, consistency protocol, UAV formation control

## Abstract

Single UAVs have limited capabilities for complex missions, so suitable solutions are needed to improve the mission success rate, as well as the UAVs’ survivability. A cooperative multi-UAV formation offers great advantages in this regard; however, for large and complex systems, the traditional control methods will be invalid when faced with unstable and changing environments. To deal with the poor self-adaptability and high requirements for the environmental state information of traditional control methods for a multi-UAV cluster, this paper proposes a consistent round-up strategy based on PPO path optimization to track targets. In this strategy, the leader is trained using PPO for obstacle avoidance and target tracking, while the followers are expected to establish a communication network with the leader to obtain environmental information. In this way, the tracking control law can be designed, based on the consistency protocol and the Apollonian circle, to realize the round-up of the target and obstacle avoidance. The experimental results show that the proposed strategy can achieve the round-up of the target UAV and guide the pursuing multi-UAV group to avoid obstacles in the absence of the initial detection of the target. In multiple simulated scenarios, the success rates of the pursuit multi-UAV cluster for rounding up the target are maintained above 80%.

## 1. Introduction

Currently, with the development of science and technology, UAVs have been widely used in military, industrial, agricultural, and other fields. However, when faced with the requirements of target search, target pursuit, and target round-up abilities, a single UAV often suffers from various problems, including an inefficient detection range and a weak ability to adapt to the environment. Thus, it is increasingly important to study the techniques involving multi-UAVs for dealing with automatic collaborative tasks. At present, many countries have been involved in the research of multi-UAV formation, including some specific military plans. As early as in 2008, the University of Pennsylvania verified the indoor formation flight and obstacle avoidance of 16–20 small quadcopters. In recent years, several low-cost multi-UAV formation projects, such as the Defense Advanced Research Projects Agency’s Pixie Project and DARPA’s Attack UAV Swarm Tactics Project from U.S., have been launched [1]. The ability to continuously traverse and monitor the specific target areas is the main advantage of multi-UAV. Due to limitations in operational accuracy and capability, a single UAV generally experiences difficulty performing tasks independently, and a multi-UAV cluster can effectively compensate for this deficiency. However, for a complex cluster system, a multi-UAV cluster must exhibit the ability to self-organize and adapt. In previous research regarding multi-agent control methods, agents were generally required to make corresponding decisions based on environmental information and individual information within the cluster. The overreliance on this information will lead to poor environmental adaptability; therefore, enabling a cluster to accomplish tasks based on less information and improving the efficiency of information utilization are current research hotspots.

The method of control of the UAV cluster mainly depends on the multi-agent system’s collaborative control techniques [2,3,4]. In 1986, Reynolds first proposed the Boids model, which was based on the observations of bird swarm [5]. It assumed that the individual can perceive the information of its neighborhoods within a certain range, and the decision was made based on the three basic principles of aggregation, separation, and alignment [6]. On this basis, Vicsek established a plane model of a discrete time system in 1995 [7] to simulate the consistent behavior of particle emergence. The above classic models have laid the foundation for traditional cluster control methods. Up until now, the formation control methods based on the principle of consistency have mainly included the leader–follower method [8,9] and the behavior control method [10]. The specific idea behind the leader–follower method is to select a leader in the cluster, with the remaining agents as the followers. The leader holds the flight path and the task’s target. In this way, based on a distributed control method, the states of the followers will gradually be consistent with the leader, and the cluster ultimately maintains stable flight. The behavior-based control method is based on the idea of swarm intelligence, according to the desired behavior pattern of the UAVs. Individual behavior rules and local control schemes are designed for each UAV, and a “behavior library” is obtained and stored in the formation controller. When the control system of the UAV is instructed, it selects and executes the behavior from the “behavior library”, according to the instruction. Based on the above general consistent control methods, Lopen improved the method for a multi-agent system [11] using visual constraints. Further, Song handled the loop formation problem with limited communication distance [12] and Hu considered the coordinated control of spacecraft formation with external interference and limited communication resources [13].

The consistent cluster control methods can achieve the collaboration of a multi-agent system, but it has the disadvantages of lacking autonomy and adaptability when facing dynamic environments and complex tasks. Therefore, from a data-driven perspective, reinforcement learning (RL) methods, which have strong decision-making capabilities [14,15], have also been widely studied in this field. Bansal [16] explored the process of generating complex behaviors for a multi-agent through a self-gaming mechanism. For dealing with problems involving discrete and continuous states in a multi-agent system, Han Hu [17] proposed the DDPG-D3QN algorithm. Jincheng [18] investigated the role of baselines in stochastic policy gradients to better apply the policy optimization methods in real-world situations. For the solutions to the offline RL problems, Zifeng Zhuang [19] found that the inherent conservativeness of policy-based algorithms needed to be overcome, and then proposed behavioral proximity policy optimization (BPPO), which did not require the introduction of any additional constraints or regularization compared to PPO. Zongwei Liu [20] proposed an actor-director-critic algorithm, which added the role of director to the conventional actor-critic algorithm, improving the performance of the agents. To address the problems of low learning speed and poor generalization in decision making, Bo Li [21] proposed PMADDPG, which is an improved version of the multi-agent deep deterministic policy gradient (MADDPG). Siyue Hu [22] proposed a noise-MAPPO algorithm, for which the success rate was over 90% in all StarCraft Challenge scenarios. Since single-agent RL exhibits the disadvantage of overestimation bias of the value function, which causes the multi-agent reinforcement learning method to learn policy ineffectively, Johannes Ackermann [23] proposed an approach that reduced this bias by using double centralized critics. Additionally, the self-attention mechanism [24] was introduced on this basis, with remarkable results. In order to improve the learning speed in complex environments, Sriram Subramanian [25] utilized the L2Q learning framework and extended the framework from single-agent to multi-agent settings. For improving the flow of autonomous vehicles in road networks, Anum [26] proposed a method based on a multi-agent RL and an autonomous path selection algorithm.

The research in the above literature has obtained certain achievements in regards to formation control and obstacle avoidance for multi-UAV. However, for these conventional algorithms, the adopted leader generally tends to follow the previously prescribed flight path. This means that if the target requiring tracked is not within the detectable range of the leader, the multi-UAV cluster cannot construct an effective decision-making mechanism, leading to failures for tracking tasks. To deal with this problem, this paper designs a consistent round-up strategy based on PPO path optimization for the leader–follower tracking problem. This strategy is based on the consistent formation control method for a leader–follower multi-UAV cluster and aims to reach the goal of target round-up and obstacle avoidance. PPO can balance the exploration-exploitation aspects, while maintaining the simplicity and computational efficiency of the algorithm’s implementation. PPO tries to avoid training instability caused by excessive updating by limiting the step size of the policy updates. This allows PPO to perform in a balanced and robust way in a range of tasks. It is supposed that each member in the multiple-UAV cluster has a detectable range for spotting the nearby target and the obstacles, and each obstacle has an impact range for causing a collision when any moving UAV enters this range. The basic principle of the proposed strategy is to force the multi-UAV cluster to approach and round-up the target, based on the consistent formation control when any member locates it, and there are no obstacles nearby, while optimizing the policy for the leader, based on PPO, to determine the best flight path and make the followers cooperated with the leader in other conditions. To verify the performances of the proposed strategy in different environments, four scenarios are considered in the numerical experiments, including environments with a fixed target, a moving target, a fixed target and a fixed obstacle, as well as a fixed target and a moving obstacle. The results showed that the strategy exhibits excellent performance in tracking the target and successfully avoiding obstacles. In summary, the main contributions of this paper can be concluded as follows:

(1) Designing a flight formation based on the Apollonian circle for tracking the target, and executing the collaborative flight of multi-UAV, based on consistent formation control, achieving the round-up for the target in situations where the target is within the detectable range and in which none of the pursuit UAVs enter the impact range of any obstacle.
(2) Optimizing the acting policy of the leader based on the PPO algorithm for finding the best flight path to track the target and avoid obstacles, achieving the round-up for the target with the help of consistent formation control in situations where the target is out of the detectable range and any of the pursuit UAVs enter the impact range of any obstacle.
(3) Validating and analyzing the performance of the proposed algorithm in regards to target round-up and obstacle avoidance in environments with a fixed target, a moving target, a fixed target and a fixed obstacle, as well as a fixed target and a moving obstacle.

The rest of this paper is designed as follows: Section 2 introduces the necessary preliminaries related to this paper; Section 3 illustrates the design principles, implementation process, and extensibility of the proposed strategy; Section 4 details the numerical experiment, and the results from the proposed round-up strategy, applied in different environments, are compared and analyzed; while Section 5 presents the conclusions.

## 2. Background

### 2.1. Leader–Follower Model of Multi-UAV Based on Algebraic Graph Theory

**Figure 1:**
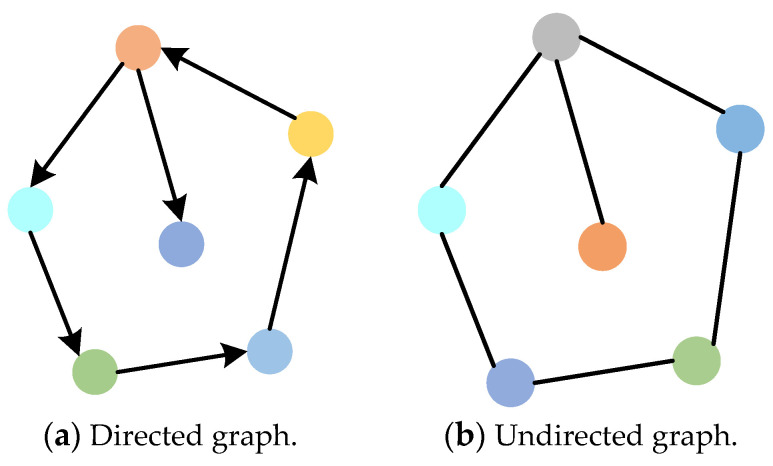
Schematic diagram of directed and undirected graphs.

**Figure 2:**
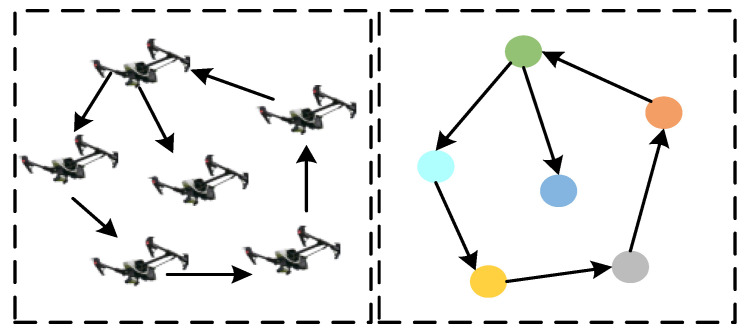
The multi-UAV cluster and its corresponding topology network.

In the modeling for a multi-UAV cluster with a leader–follower structure, it is noted that the leader needs to send the information, including its position and attitude, to all followers at a certain time point [27]. Therefore, in order to achieve information sharing among the cluster individuals, it is necessary to establish a communication topology network. Since the graph theory is an important branch of operations research, it can be widely applied to model communication relationships among cluster individuals. Therefore, the communication topology network between individual UAVs can be represented by a graph, which can be seen in Figure 1a,b. In directed graph, the information communication is one-way. In undirected graph, the information exchange can be two-way. This paper applies a directed graph to model a leader–follower multi-UAV cluster, and the corresponding topology network is shown in Figure 2. The information exchange of members of the leader-follower multi-UAV cluster is supposed to be one-way.

For the involved multi-UAV cluster, let G=(V,E,A) denote the directed graph to represent the topological structure for the cluster. In the graph, G***,*** V is the set of vertices, E ***(***E⊆V×V***)*** is the set of edges $e_{ij}=(i,j)$, which represents the path that exists between node $v_{i}$ and node $v_{j}$, and A represents the weighted adjacency matrix of the graph G. For the arbitrary nodes $v_{i}$ and $v_{j}$, it is noted that the graph is strongly connected if there edge $e_{ij}$ and $e_{ji}$ exist. On the contrary, if only one edge exists for nodes $v_{i}$ to $v_{j}$*,* the graph becomes fully undirectedly connected.

The adjacency matrix $A=[a_{ij}]\in R^{n\times n}$ represents the adjacency relationship between the vertices in the graph, and it is two-dimensional, where $a_{ij}$ is a non-negative real number that represents the weight of the edge between node $v_{i}$ and node $v_{j}.$ For the directed graph, if an edge exists between node $v_{i}$ and node $v_{j}$, $a_{ij}$ is recorded as 1; otherwise, it is 0. Moreover, for the undirected graph, it satisfies $a_{ij}=a_{ji}$. Therefore, the expression of $a_{ij}$ is as follows:

(1) $$a_{ij}=\left\{\begin{array}{c} 1,\;\;\;\;\;\;\;(v_{i},v_{j})\in V\\ 0,\;\;\;\;\;\;\;\;\;\;\;\;\;\;\;others \end{array}\right.$$

In addition to the adjacency matrix A, the Laplace matrix ***L***(***G***) is defined to describe the characteristic of the graph:

(2) $$L=[l_{ij}]=D-A$$

where $D=diag\left\{d_{0},d_{1},\cdots ,d_{n}\right\},\;\in R^{n\times n}$ is a diagonal matrix, and the element $d_{i}$ can be calculated according to the following equation

(3) $$d_{i}=\sum_{j=1}^{n}a_{ij},\;i=1,2\ldots n$$

Therefore, the matrix ***L***(***G***) is an asymmetric matrix, whose rows sum to zero.

**Lemma** **1** **[28].** *Denote **G** as the graph which contains n nodes, and the Laplace matrix associated with the graph **G** is *$L={\left[l_{ij}\right]}^{n\times n}$*. The Laplace matrix has zero eigenvalues with the algebraic weight 1, and the rest of the eigenvalues have positive real parts. Denote these eigenvalues as* $\lambda_{1}(L),\lambda_{2}(L),\ldots ,\lambda_{n}(L)$*, then these eigenvalues satisfy:*

(4) $${R(\lambda }_{1}(L))<R(\lambda_{2}(L))\leq \cdots \leq R(\lambda_{n}(L))$$

*where*$R(\lambda_{n}(L))$ *represents the real part of eigenvalue* $\lambda_{n}(L)$*. When there is an information exchange in the communication topology network, the eigenvalue real part of the Laplace matrix corresponding to the graph will be or greater than 0.*

In the applied multi-UAV leader–follower model, the individual UAVs are considered as vertices of the graph, and the presence or absence of edges represents the existence of an information exchange between the UAVs. For a particular UAV, if the neighbors appear within the communication acceptance range of itself, the corresponding edges will be generated in the graph. In this paper, two communication topology networks will exist, including the one which contains the members of the pursuit multi-UAV cluster and the other which contains the members of cluster and the target.

### 2.2. Description of the Proximal Policy Optimization Algorithm

A multi-UAV cluster can round up the target when this target is in the detectable range of any pursuit UAV. However, in potential bad conditions, the multi-UAV cluster is unable to detect the target at the initial moment. This will cause the adjacency matrix of the graph to become a zero matrix, thus leading to the failure of the task. To solve this problem, we introduce the PPO algorithm for the leader to guide the followers to find the target when the cluster cannot detect it initially, thus realizing the goal of avoiding obstacles and rounding up the target.

#### 2.2.1. Policy Gradient

The PG algorithm is a policy-based reinforcement learning algorithm. The algorithm represents the strategy as a continuous function related to the reward function. The continuous function optimization method is then used to find the optimal strategy, and the optimization objective is to maximize the continuous function [29,30].

The objective function can be assumed to be J(θ), and a parameterized policy function $\pi_{\theta }(s,a)=P[a|s,\theta ]$ is obtained by neural network training to obtain the highest reward, so it is necessary to find an array of parameters which can create the best J(θ). This is usually achieved by using the gradient descent method. Thus, the process of updating the parameters can be represented as follows:

(5) $$\nabla_{\theta }J(\theta )=E_{\pi_{\theta }}[\nabla_{\theta }\log\pi_{\theta }(s,a)Q_{\pi }(s,a)]$$

(6) $$\theta =\theta +\alpha \nabla_{\theta }J(\theta )$$

Then, the strategy will obtain the trajectory which can maximize the mean of the reward.

#### 2.2.2. Proximal Policy Optimization

PPO is a policy gradient (PG) method of a reinforcement learning algorithm, in which the core idea is to adjust the probability of the sampling actions, thus achieving an optimized policy based on good return. Generally, the objective function for a PG algorithm can be written as:

(7) $$L^{PG}(\theta )={\hat{E}}_{t}[log\pi_{\theta }(a_{t}|s_{t}){\hat{A}}_{t}]$$

where $\pi_{\theta }$ represents the policy, ${\hat{A}}_{t}$ represents the estimate of the advantage function at time step *t*, and ${\hat{E}}_{t}$ represents the average empirical value of a finite batch of samples. The policy parameter θ can be optimized using a stochastic gradient descent method. On the basis of this on-policy algorithm, the application of two different policies can be considered to improve the sampling efficiency and transform the algorithm in an off-policy way. Thus, in PPO, it is denoted that $\pi_{\theta }(a_{t}|s_{t})$ represents the policy which interacts with the environment, and $\pi_{\theta old}(a_{t}|s_{t})$ represents the strategy that is updated in an inner-looped manner. Further, the learning goal can be revised into:

(8) $$\underset{\theta }{\mathrm{maximize}}{\hat{E}}_{t}[\frac{\pi_{\theta }(a_{t}|s_{t})}{\pi_{\theta_{old}}(a_{t}|s_{t})}{\hat{A}}_{t}-\beta KL[\pi_{\theta_{old}}(\cdot |s_{t}),\pi_{\theta }(\cdot |s_{t})]]$$

where KL is the Kullback–Leibler divergence, which is used to limit the distribution difference between $\pi_{\theta }$ and $\pi_{\theta old}$, and β is a positive penalty factor that dynamically adjusts the function of KL.

In addition, another version of PPO, called PPO2, limits the updating progress by means of truncation. In PPO2, the objective function can be written as:

(9) $$L^{CLIP}(\theta )={\hat{E}}_{t}[min(r_{t}(\theta ){\hat{A}}_{t},clip(r_{t}(\theta ),1-\varepsilon ,1+\varepsilon ){\hat{A}}_{t})]$$

where ε denotes the truncation hyperparameter, which is generally set around 0.2, and clip denotes the truncation function, which is responsible for limiting the proportion to ensure the convergence.

## 3. A Consistent Round-Up Strategy Based on PPO Path Optimization

**Figure 3:**
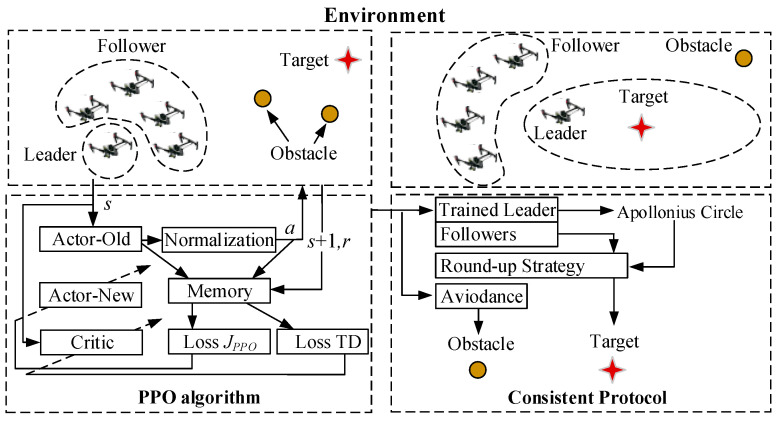
Diagram of the proposed consistent round-up strategy, based on PPO path optimization.

The traditional formation control method for a multi-UAV cluster generally requires leaders follow a previously prescribed flight path, which may lead to the failure of target tracking and obstacle avoidance when faced with a complex environment. Specifically, when based on the consistency protocol only, the round-up mission will fail if the pursuit multi-UAV cluster cannot detect the target, or if the round-up route is interrupted by an obstacle. To deal with this problem, this paper designs a consistent round-up algorithm based on PPO path optimization for the leader–follower [31] tracking problem, as shown in Figure 3.

The proposed strategy assumes that one leader and several followers exist in the multi-UAV cluster. From Figure 3, it can be noted that the proposed round-up strategy consists of two main parts: the PPO algorithm and the consistency protocol. The leader is trained and controlled by the PPO algorithm, playing the role of tracking the target and avoiding the obstacles in the environment when the target is out of the cluster’s detectable range. the PPO-based reinforcement learning algorithm will plan the optimal flight path of the leader. Once the optimal flight path is maintained, the followers are expected to follow the leader through the consistency protocol. When the target can be detected by the cluster, the consistency protocol will control the cluster to round up the target, based on the formation of an Apollonian circle. The strategy combines the two parts above to guide the cluster to finish the mission of safely rounding up the target.

### 3.1. Discrete-Time Consistency Protocol

The purpose of this cluster is to simultaneously round up a single target and avoid obstacles. The area in which the target can be detected is defined as the “detectable area”. If none of the individuals in this pursuit cluster can detect the target, the leader needs to plan a flight path to approach the detectable area of the target. Once the leader is able to enter the detectable area, the round-up path can be planned based on the Apollonian circles, which requires a cooperative flight according to a consistent protocol.

For a two-dimensional discrete-time system, considering that there are N UAVs in the cluster, the dynamics for each individual can be described by the following model:

(10) $$r_{i}(k+1)=r_{i}(k)+Tv_{i}(k)$$

(11) $$v_{i}(k+1)=v_{i}(k)+Tu_{i}(k)$$

where i=1,2,…,N, $r_{i}(k)\in R^{n}$,  $v_{i}(k)\in R^{n}$, and $u_{i}(k)\in R^{n}$ represent the position, velocity, and control inputs of the ith member, respectively. Moreover, T denotes the sampling period (T>0). For any i,j=1,2,…,N, if the system in any initial state satisfies the following conditions:

(12) $$\underset{k\to \infty }{\lim}\|x_{i}(k)-x_{j}(k)\|=0$$

(13) $$\underset{k\to \infty }{\lim}\|v_{i}(k)-v_{j}(k)\|=0$$

then the discrete system is capable of achieving asymptotic consistency.

Assume that each member can only communicate with the adjacent neighbors in its communication region, and the set of adjacent neighbors $N_{i}^{\alpha }$ for the ith member at moment k can be expressed as:

(14) $$N_{i}^{\alpha }(k)=\{j:||x_{i}-x_{j}||\leq r,j=1,2,\ldots ,N,j\neq i\}$$

where α indicates the communication topology network of the pursuit cluster, ||·|| denotes the Euclidean distance, and r is the maximum communication radius between the ith and jth member. It is noted that the cluster should eventually reach a consistent state when performing the tracking task, which means that the direct distance between the individual members should be maintained, as required. Therefore, based on the definition of asymptotic consistency of the discrete system, the following constraint must be satisfied for the cluster:

(15) $$\left||x_{i}-x_{j}||=d,\forall i,j\in N_{i}^{\alpha }(k\right)$$

where *d* is the required distance between neighboring UAVs in a consistent steady state.

Thus, based on the consistency protocol, the control law $u_{i}$ for the ith member in the multi-UAV cluster can be composed of three components, as follows [32]:

(16) $$u_{i}=u_{i}^{g}+u_{i}^{d}+u_{i}^{\gamma }$$

where $u_{i}^{g}$ controls the safety distance among the cluster members; $u_{i}^{d}$ controls the consistent speed for the cluster members, and $u_{i}^{\gamma }$ control the pursuit UAV to achieve the same speed as the target and maintain the round-up distance based on the Apollonian circle. The specific definitions for $u_{i}^{g}$***,*** $u_{i}^{d}$, and $u_{i}^{\gamma }$ are as follows:

(17) $$u_{i}^{g}=k_{\alpha }\sum_{j\epsilon N^{i}}^{n}a_{ij}[\|r_{i}-r_{j}\|-d]$$

(18) $$u_{i}^{d}=k_{d}\sum_{j\epsilon N^{i}}^{n}a_{ij}[(v_{i}-v_{j})]$$

(19) $$u_{i}^{\gamma }=k_{\gamma }\sum_{i\epsilon N^{\rho }}^{n}a_{\rho j}\{[\|r_{\rho }-r_{i}\|-dc]+(v_{\rho }-v_{i})\}$$

From Equations (17)–(19), the coefficients $k_{\alpha }$, $k_{d}$, and $k_{\gamma }\;$ represent the control parameters, $N^{i}$ indicates the set of neighbors in the communication adjacency network, and $a_{ij}$ denotes the elements in the adjacency matrix. When other members appear within the detection range of the ith member, a communication path between the ith member and the neighbor will be quickly established. In this way, the corresponding element of the adjacency matrix is $a_{ij}=1$; otherwise,$\;a_{ij}=0$. Additionally, $N^{\rho }$ represents the set of neighbors in the communication adjacency network and the target. If there is a member which has discovered the target, $a_{\rho j}$ is set as 1; otherwise, $a_{\rho j}=0$. The symbol $r_{i}=(x_{i},y_{i})$ indicates the position coordinates, and $v_{i}=(v_{xi},v_{yi})$ indicates the velocity coordinates of the ith member in the inertial coordinate system. The symbol ρ represents the target UAV, *d* is the safe distance between the pursuing UAVs, and *dc* denotes the capture distance required in the round-up task.

From the descriptions from Equations (17)–(19), it is concluded that $u_{i}^{g}$ induces the separations of the members in the cluster so that the minimum distance between the members can be maintained; $u_{i}^{d}$ causes the speed alignment of the pursuit UAVs to maintain a consistent speed; and $u_{i}^{\gamma }$ elicits the alignment of the pursuit UAVs with the speed and relative distance of the target, realizing the round-up of the target.

### 3.2. Target Round-Up Based on the Apollonian Circle

When the leader enters the detectable area of the target, the cluster needs to surround the target and round it up. To achieve this goal, it is necessary to design a round-up route based on the Apollonian circle [33]. Rounding up the target with multiple UAVs ensures, to the greatest extent, that the target cannot escape after being tracked. In order to simplify the formation design process, it is assumed that the speed values of the UAVs do not change during the task.

**Figure 4:**
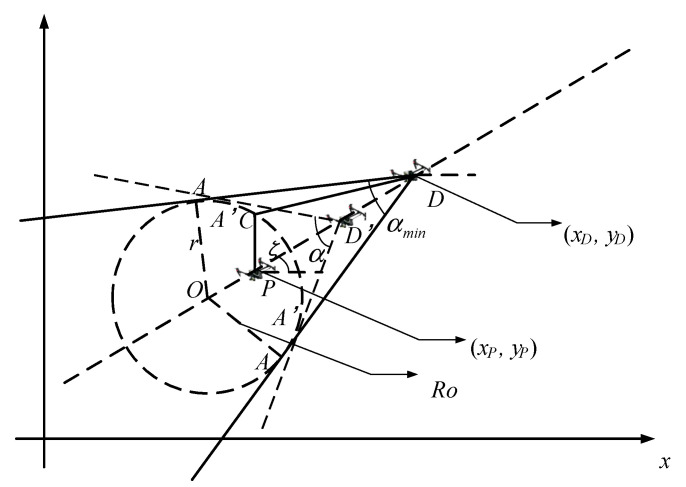
Diagram of an Apollonian circle.

The diagram of an Apollonian circle is drawn in Figure 4. Suppose that point P is the position of a pursuit UAV and its velocity is ${\;v}_{p}$, and point *D* is the position of the target and its velocity is $v_{D}$; then the ratio k is expressed as follows:

(20) $$k=v_{P}/v_{D}$$

The circle shown in Figure 4 is the so-called Apollonian circle, where *O* is the center, and $R_{o}$ is the radius. The position of center *O* and the radius $R_{o}$ can be expressed as [34]:

(21) $$x_{O}=\frac{x_{D}-x_{P}k^{2}}{1-k^{2}},y_{O}=\frac{y_{D}-x_{P}k^{2}}{1-k^{2}}$$

(22) $$R_{O}=\frac{k\rho }{1-k^{2}}$$

where $\rho =\sqrt{{\left(x_{P}-x_{D}\right)}^{2}+{\left(y_{P}-y_{D}\right)}^{2}}$ represents the distance between point *D* and point *P*.

From Figure 4, it is seen that *C* is an arbitrary point located on the Apollonian circle. Define the angle of the tangent line between the target and the Apollonian circle as α. In the case where the ratio of the target velocity to the pursuing UAV’s velocity is k, the pursuit UAV will not be able to successfully pursue the target when the angle of the tangent line between the target and the Apollonian circle is less than $\alpha_{min}$, which can be expressed as follows:

(23) $$\alpha_{min}=2\arcsin\left(\frac{V_{p}}{V_{D}}\right)=2\arcsin\left(k\right)$$

It can be seen that when the angle α is greater than $\alpha_{min}$, the UAV P can always find an angle ζ that is able to catch the target.

**Figure 5:**
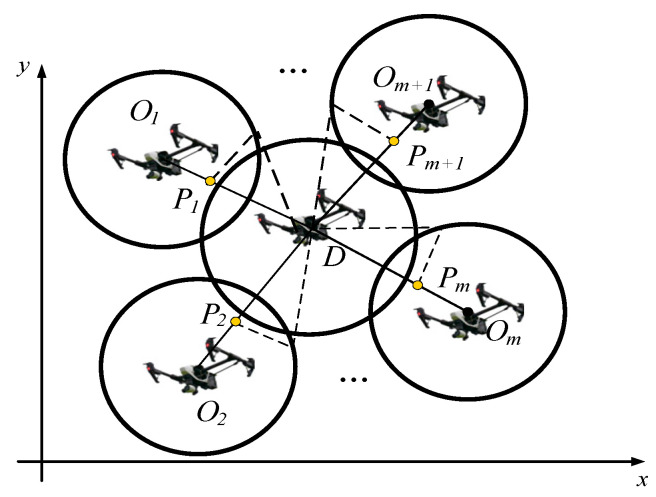
Diagram of the round-up formation of the pursuit cluster.

Therefore, when multiple pursuit UAVs are employed, the cluster can form several Apollonian circles to surround the target, thus the rounding up target by the pursuit cluster and preventing its escape. To achieve this round-up goal, it is always desirable that the target should be within the Apollonian circles formed by all of the pursuing UAVs, as shown in the Figure 5.

**Figure 6:**
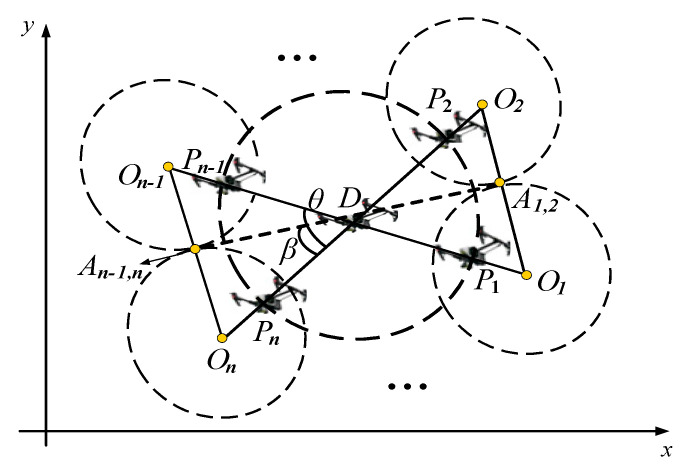
The final formation of the round-up condition.

Uses *D* to represent the target to be rounded up and $O_{i}$ to represent the center of the Apollonian circle formed by the ith pursuit UAV and the target. The details of the formed Apollonian circle can be obtained based on Equations (20)–(23). In order to round up the target, it is necessary to design the desired position $P_{i}$ for each pursuit UAV. In this way, when the pursuit UAV can detect the target, it will continuously fly towards $P_{i}$, thus completing the round-up for the target. The final formation of the round-up condition is shown in Figure 6.

In Figure 6, $A_{n-1,n}$ represents the tangent point formed by the (n−1)th and nth Apollonian circles. Denote the angle formed by the points of any adjacent centers of the Apollonian circle and the center D as θ, and denote the angle formed by the points between the center of any Apollonian circle and the corresponding tangent point as β; then, it is seen that θ=2β. Combining the geometric properties and the definition of an Apollonian circle, we can obtain the following relationships:

(24) $$k=v_{P}/v_{D}=\frac{O_{n}A_{n-1,n}}{OA_{n-1,n}}=\frac{O_{n-1}A_{n-2,n-1}}{OA_{n-2,n-1}}=\cdots =\frac{O_{1}A_{1,2}}{OA_{1,2}}=\sin\beta$$

(25) $$\theta =2\beta =2\sin^{-1}k=2\sin^{-1}\frac{v_{P}}{v_{D}}$$

Based on the above designed formation, it can be seen that if the position of the leader $\left(x_{1},y_{1}\right)$ is known, then the designed positions $\left(x_{i},y_{i}\right)$ of the followers can be known as:

(26) $$x_{i}=x_{1}+Rsin(i\theta )$$

(27) $$y_{i}=y_{1}-Rcos(i\theta )$$

(28) dc=R=ρ

where i=1,⋯,N−1, and dc is the capture distance required in the round-up task. To ensure that the formed Apollonian circles can closely surround the target, the minimum distance d between neighboring pursuit UAVs can be set as follows:

(29) d=2dc∗sin⁡β

Thus, the target is expected to be rounded up by the pursuit cluster, and this round-up strategy is used as a basic strategy for the multi-UAV cluster when the target can be detected, and there are no obstacles nearby.

### 3.3. Target Tracking and Obstacle Avoidance Based on the Proposed Strategy

**Figure 7:**
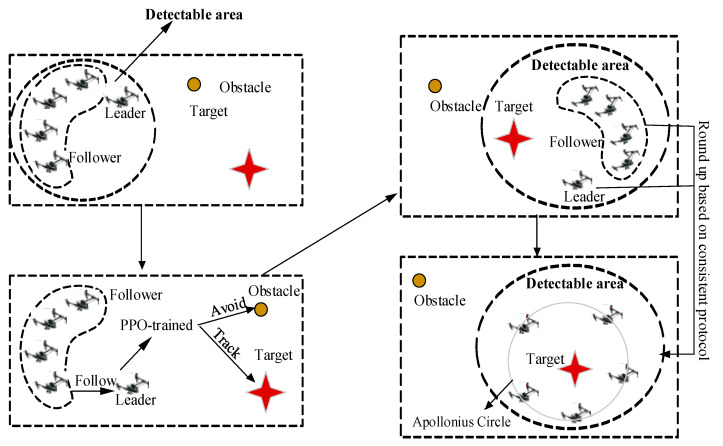
The process of the proposed round-up strategy.

The whole process is shown in Figure 7, where we use a circle to express the obstacle and a star to represent the target. It is seen that for situations in which the leader is able to reach the detectable area of the target, the target can be rounded up based on the round-up route and consistency protocol provided in Section 3.1 and Section 3.2. However, when facing a complex environment in which the leader is unable to directly reach the detectable area of the target, or where certain obstacles exist, it is necessary to plan an optimal flight path for the leader. The optimization process is conducted based on the PPO algorithm. By using such a reinforcement learning method, the leader can be guided to reach the detectable area in an optimized way, thereby further completing the encirclement of the target by other followers.

**Figure 8:**
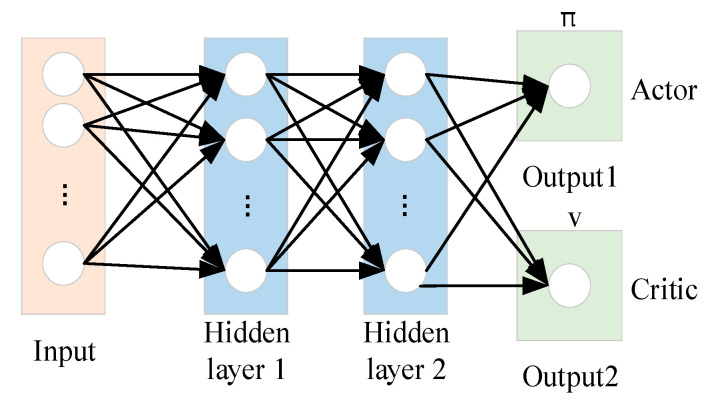
Diagram of PPO network.

The PPO algorithm consists of two types of networks, including the actor network and the critic network. With input which contains the states of the leader, the target, and the obstacle, the actor networks can generate the corresponding policies and the corresponding actions, and the critic network generates the state value function. The whole diagram of the actor network and the critic network can be shown in Figure 8.

In Figure 8, the input layer has six input nodes: [$x_{leader}$, $y_{leader}$, $x_{target}$, $y_{target}$, $x_{obstacle}$, $y_{obstacle}$], where [$x_{leader},y_{leader}$] represents the position of the leader itself, [$x_{target},y_{target}$] represents the relative position of the target, and [$x_{obstacle},y_{obstacle}$] represents the relative position of the obstacle. Then, the activation layer are selected as the type of ReLU functions. After that, there are two fully connected layers, comprising 256 cells. The output layer of the actor network possesses two nodes corresponding to the amount of change in the horizontal coordinates $∆x_{leader}$ and the amount of change in the vertical coordinates $∆y_{leader}$ of the leader. The output layer of the critic network is designed as one node, corresponding to the state value function.

**Figure 9:**
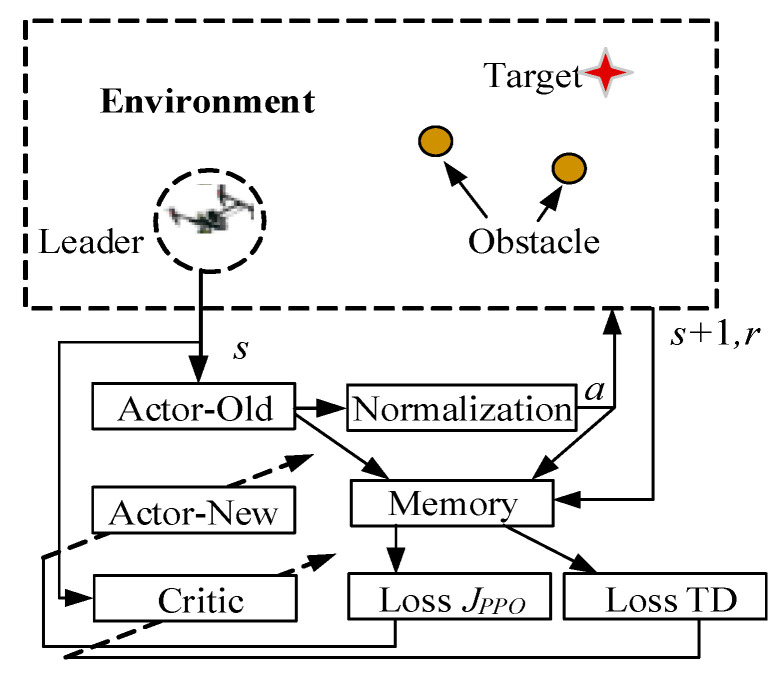
Diagram of algorithm network updates.

The network’s PPO update process is shown in Figure 9. The first step is to initialize the conditions of the target, leader, and obstacle, where their position and speed are randomly generated within a certain range. Then, the relative state $s_{t}$ can be calculated and inputted into the PPO algorithm. Based on the policy network, the leader’s action $a_{t}$ will be outputted and executed to the environment. After the interaction, the next state $s_{t+1}$ and the reward $r_{t}$ can be obtained. To repeat the above steps, the trace $\left\{s_{0},a_{0},r_{0},s_{1},a_{1},r_{1},...,s_{T-1},a_{T-1},r_{T-1}\right\}$ can be obtained and then stored in the memory.

Based on the trajectory $\tau =\left\{s_{0},a_{0},r_{0},s_{1},a_{1},r_{1},\cdots ,s_{T-1},a_{T-1},r_{T-1}\right\}$ from the memory, it is possible to obtain the state value function $V(s_{t})$. To ensure that the output of the critic $V_{pre}(s_{t})$ is close to $V(s_{t})$, the loss function for the critic network can be expressed as follows:

(30) $$L_{Critic}=\frac{1}{T}\sum_{t=0}^{T-1}{\left[V_{pre}(s_{t})-V(s_{t})\right]}^{2}$$

As for the actor network, the loss function is shown in Equation (9) in Section 2.2. Through the descent gradient method, the network parameters in the actor and critic networks can be updated to cause the leader to approach the target and better avoid the obstacles.

## 4. Experimental Results

This section presents the experimental results for the proposed consistent round-up strategy. First, the experimental environment setting is introduced. Then, the analysis of performance regarding the consistency protocol and the PPO path optimization in the proposed strategy are conducted, respectively. Additionally, the generalization ability of the proposed strategy is verified.

### 4.1. Experimental Environment Setting

The experiments are performed in a two-dimensional airspace environment. The training hyperparameters, environmental parameters, and function parameters are shown in Table 1, Table 2 and Table 3, respectively. Among them, the training of the leader is based on the PTYORCH framework, and the display of the cluster and the target is carried out based on the MATLAB platform.

**Environment.** This paper employs a two-dimensional environment of multi-UAV clusters based on continuous observation information and discrete action space, which can be divided into the target round-up environment and the leader’s training environment. The former environment can include the target, obstacle, and multi-UAV cluster with a trained leader, while the latter environment can include the leader, target, and obstacle. Among these, the role of the multi-UAV cluster is to round up the target. The role of the leader is to lead the cluster to avoid the obstacle and track the target out of the detectable range, and the target will escape when it locates any pursuit UAV. If the leader hits an obstacle, it will receive a minus 100 bonus value, and if the leader completes the obstacle avoidance and the leading task, it will earn a positive 100 bonus value. The reward design and escape strategy for the target is detailed in Appendix A and Appendix B.

**Leader Training Environment.** This environment is divided into four specific cases, including the one with no obstacle, but a fixed target; the one with no obstacle, but a moving target; the one with a fixed target and a fixed obstacle; and the one with a fixed target and a moving obstacle. The leader aims to earn more rewards by avoiding collisions with the obstacle and reaching the detectable area of the target as soon as possible. Based on the PPO algorithm, the path of the leader will be optimized.

**Target Round-Up Environment.** This environment is divided into two cases. The first case is the one in which there is no obstacle in the environment and the cluster can detect the target at the initial moment; the cluster will round up the target based on the consistency protocol proposed in Section 3.1. The second case is the one in which an obstacle exists in the environment, and the cluster cannot detect the target at the initial moment; the trained leader will guide the followers for obstacle avoidance and target tracking. When the cluster reaches the detectable area of the target, it will then round up the target, based on the consistency protocol.

To evaluate the effectiveness of the strategy, the success rate of the round-up task $S_{r}$ is defined and expressed as

(31) $$S_{r}=dc/\left(\frac{\sum_{i=1}^{N}d_{i}}{N}\right)$$

where dc is the capture distance, N is the number in the multi-UAV cluster, and $d_{i}$ is the distance between the ith pursuit UAV and the target.

### 4.2. Experiment Using the Round-Up Strategy Based on Consistency Protocol

**Figure 10:**
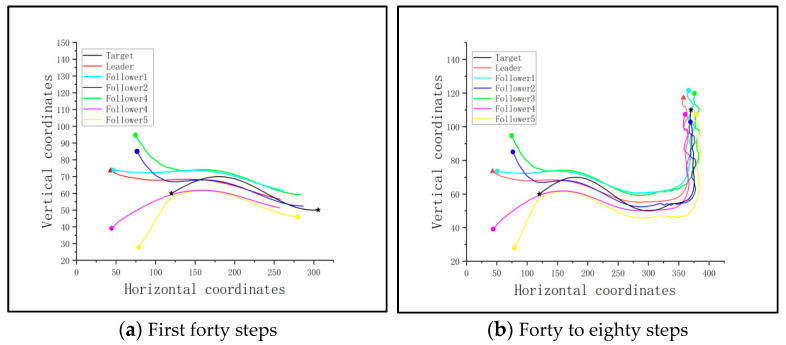
The traces of the pursuit cluster and the target using the method of consistency protocol: no obstacle and a fixed detectable target.

In this scenario, it is considered that the multi-UAV cluster takes the round-up strategy, based on a consistency protocol. It supposes that the target can be detected by the cluster, and there is not any obstacle nearby. The involved pursuit cluster includes one leader and five followers, and there is one target that needs to be captured. The length of the time step is set to 0.05 s, and the number of simulated steps is set to 80. The target is set to follow a route of y=10∗sin⁡t+60 before it finds any pursuit UAV. The round-up formation of the cluster is based on the Apollonian circles designed in Section 3.2, and the consistency protocol is adopted during the mission. In this way, the traces of the pursuit cluster and target are shown in Figure 10, where the black star represents the target, the triangle represents the leader, and the others are followers.

After 80 steps of the flight, the distance between each pursuit UAV and the target is shown in Table 4.

In Table 4, $d_{l}$ represents the distance between the leader and the target, and $d_{fi}$ represents the distance between the ith follower and the target. In this case, $S_{r}$ equals 90.10%, which indicates that the multi-UAV cluster has a 90.10% success rate in regards to the round-up task for the target.

**Figure 11:**
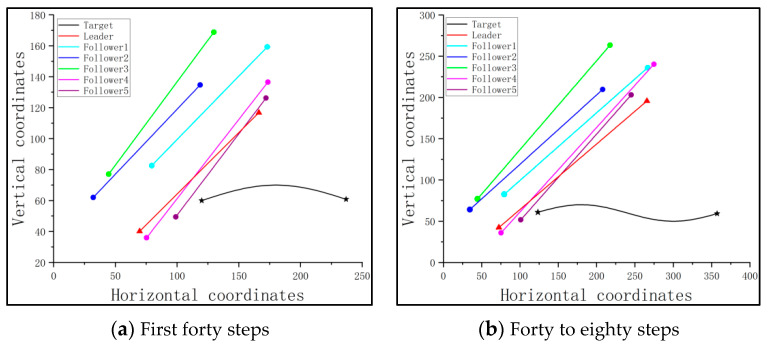
The traces of the pursuit cluster and the target with the method of consistency protocol: no obstacle and a fixed undetectable target.

It is seen that the cluster performs well based on the consistency protocol when the target can be initially detected. However, if the cluster loses the target at the initial moment, it will likely lead to a failure. Set the detectable range of the cluster to be reduced to 1 m, and the target will be out of the cluster’s detectable range. The relevant traces of the cluster and the target are shown in Figure 11.

It is seen that in this condition, the adjacency matrix in the communication topology for the cluster will be 0; thus, the cluster cannot complete the round-up mission.

### 4.3. Experiment of Consistent Round-Up Strategy Based on PPO Path Optimization

Considering the condition that the target is not within the detectable range of the pursuit cluster, or obstacles exists nearby, the leader should be trained to choose the optimized flight path. The followers are expected to be guided by the leader and round up the target.

**Figure 12:**
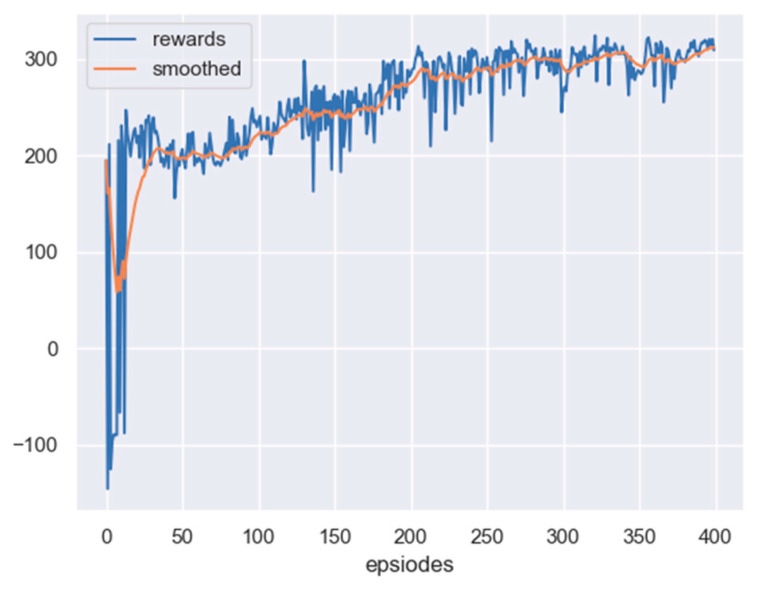
The reward curve of the leader in PPO training: no obstacle and an undetectable fixed target.

The scenario considered here is the one with no obstacle, but a fixed target. The initial position of the leader is set as $\left(240,415\right)$ m, and the initial position of the target is set as $\left(300,300\right)$ m. Based on the PPO algorithm, the reward curve, along with learning episodes, is shown in Figure 12. The curve will also be smoothed by the moving average method. From the figure, it can be seen that the reward of the leader has been improved, which indicates that the leader can reach the detectable area of the target.

**Figure 13:**
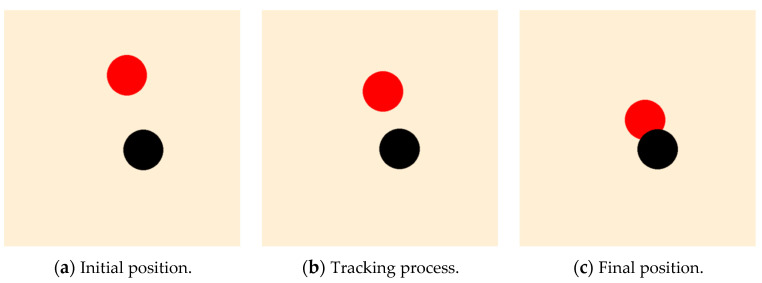
The diagram of the leader after PPO training: no obstacle and an undetectable fixed target.

The display effect of the trained leader is shown in Figure 13. In the figure, the red and black circles represent the leader and the target, respectively, and the radius of the circle shows the relevant physical body. From Figure 13a–c, it is noted that after 400 training episodes, the leader can approach the target by the shortest path.

**Figure 14:**
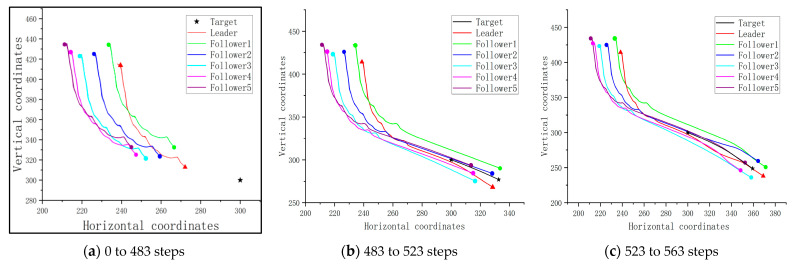
The traces of the pursuit cluster and the target with a PPO-trained leader: no obstacle, but an undetectable fixed target.

Since the followers should cooperate with the trained leader, the cluster is guided under the optimized flight path of the leader, rounding up the target based on the consistency protocol when the target is detectable. Figure 14 shows the traces of the cluster and the target of this scenario.

From Figure 14a, it is seen that the followers follow the path of the leader to approach the target at the early stage of the mission. And when the cluster reaches the detectable range of the target, they round up the target together, as shown in Figure 14b,c. The distances between the pursuit UAVs and target after 80 steps of flight, as well as the success rate, are shown in Table 5.

### 4.4. Generalization Experiment Using the Proposed Round-Up Strategy

To verify that the proposed strategy can be applied, not only to the scenario in Section 4.3, but to additional situations, this experiment simulates and analyzes the performance of the proposed consistent round-up strategy in other scenarios, including the one with no obstacle, but a moving target, the one with a fixed obstacle and a fixed target; and the one with a moving obstacle and a fixed target.

#### 4.4.1. Performance under the Scenario with No Obstacle, but a Moving Target

**Figure 15:**
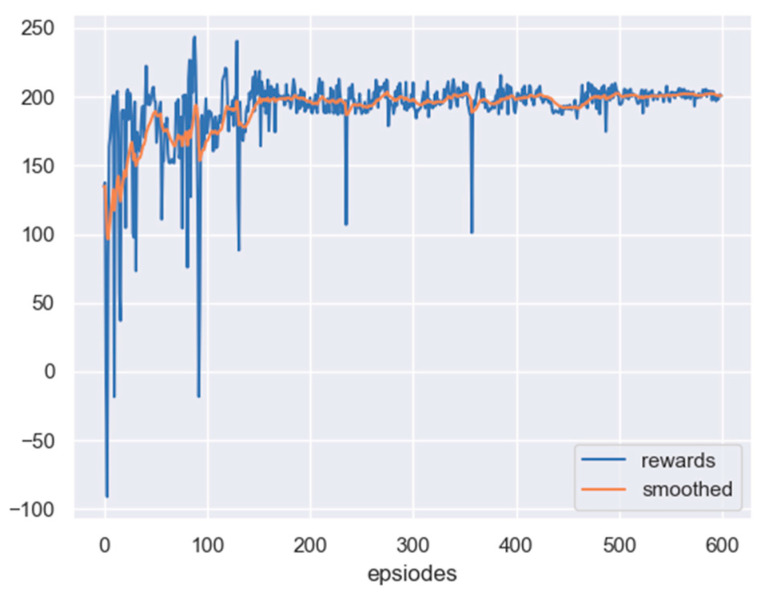
The reward curve of the leader in PPO training: no obstacle, but a moving target.

In this scenario, the initial position of the leader is set as $\left(425,450\right)$ m, and the initial position of the target is set as $\left(300,300\right)$ m. Additionally, more time steps are required to show the effect of tracking a moving target. Based on the PPO algorithm, the leader can be trained. The reward curve, along with the learning episodes, are shown in Figure 15. From the figure, it can be seen that after 600 times of training, the reward curve converges, and the leader can reach the area where it can detect the target.

**Figure 16:**
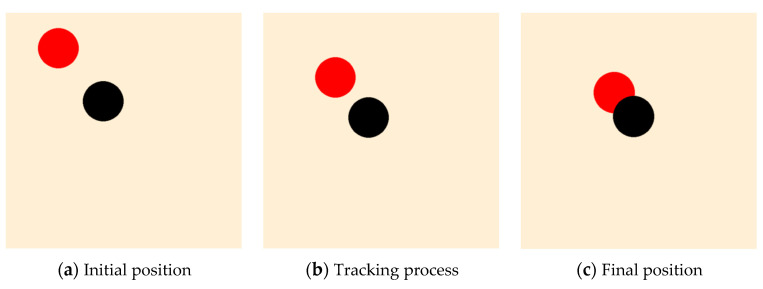
The diagram of the leader after PPO training: no obstacle and an undetectable moving target.

It can be seen from the curve that when the reward curve reaches convergence, the reward is 100 scores lower than that shown in Figure 12. The reason is that when tracking a moving rather than a fixed target, the leader will be more easily penalized because this does not always guarantee that the relative distance can continuously decrease. The process of the trained leader tracking the target is displayed in Figure 16.

**Figure 17:**
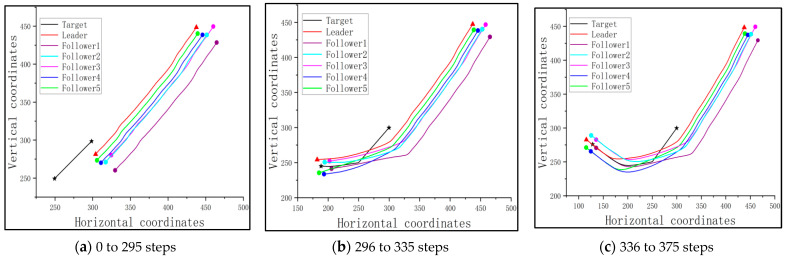
The traces of the pursuit cluster and the target with a PPO-trained leader: no obstacle, but a moving target.

Similar to the condition shown in Section 4.3, when the target cannot be initially detected, the followers in the cluster must follow the path of the leader, which can be trained based on PPO. And based on the consistency protocol, the cluster will round up the target once the leader can locate it. The relevant traces are shown in Figure 17.

From Figure 17, it can be seen that the cluster can effectively track and round up a moving target. To provide more details regarding this round-up mission, the final distances between the pursuit UAVs and the target, as well as the success rate, are shown in Table 6.

Table 6 shows that in the scenario with no obstacle, but a moving target, the success rate $S_{r}$ is 84.35%, which is slightly higher than the 82.52% obtained in the scenario with a fixed target. However, it also indicates that the maximum distance and the minimum distance have more deviations from the required capture distance, which means that the flight is not as stable as the one involving a fixed target.

#### 4.4.2. Performance under the Scenario with a Fixed Target and a Fixed Obstacle

In this experiment, the scenario includes a fixed target and a fixed obstacle, and the goal of the cluster is to round up the target while avoiding the obstacle. The initial position of the leader is set as (100,100) m, the initial position of the target is set as (400,400) m, and the initial position of the obstacle is set as $\left(300,300\right)$ m.

Based on the PPO algorithm, after 500 episodes of training, the reward curve of the leader converges, which means that the leader can reach the detectable area of the target. The corresponding reward curve is shown in Figure 17.

**Figure 18:**
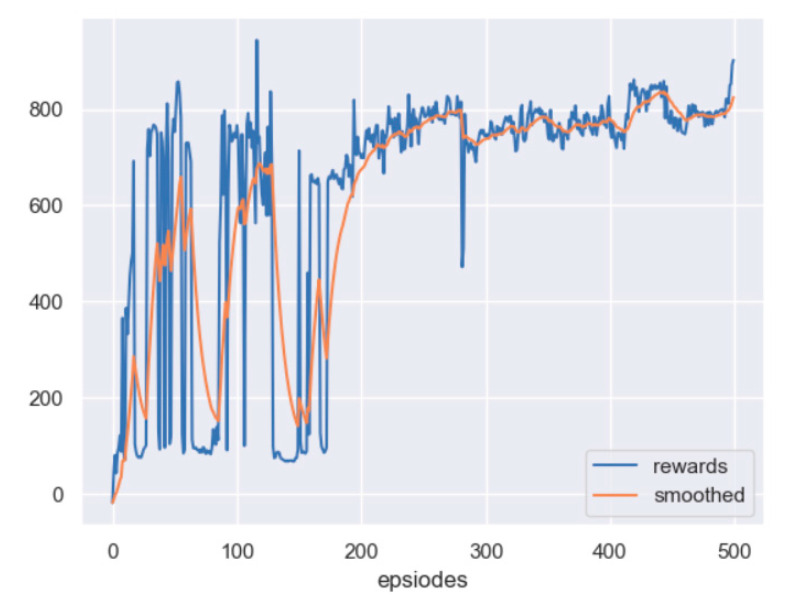
The reward curve of the leader in PPO training: a fixed target and a fixed obstacle.

**Figure 19:**
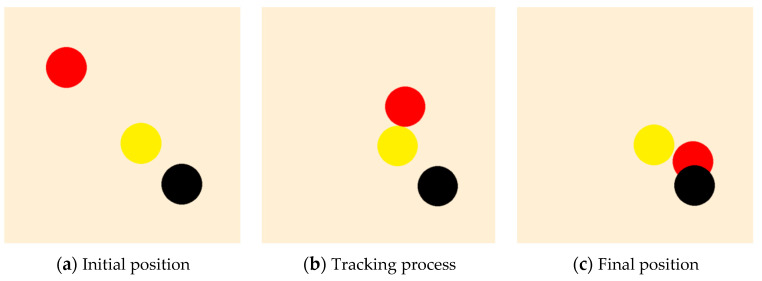
The diagram of the leader after PPO training: a fixed target and a fixed obstacle.

From Figure 18, it is observed that the leader can approach the target and avoid the obstacle after being trained, and the converged reward is about 800 scores, which is higher than that in Figure 12 and Figure 15 because of the extra bonus earned by obstacle avoidance. The display after training is shown in Figure 19, where the red circle is the leader, the black circle is the target, and the yellow circle is the obstacle. It can be seen that the leader can avoid the impact range of the obstacle by reaching the detectable range of the target after the training.

**Figure 20:**
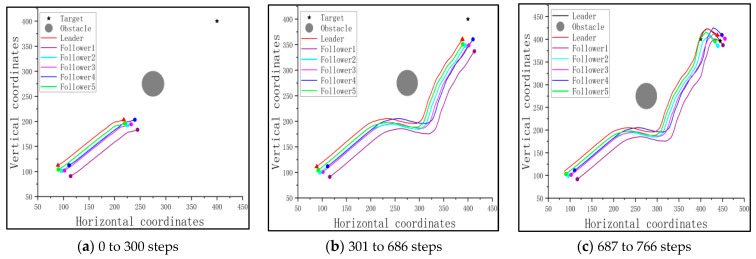
The traces of the pursuit cluster and the target with a PPO-trained leader: a fixed target and a fixed obstacle.

The traces of the leader, followers, and the target are shown in Figure 20, where the gray range represents the impact range of the obstacle.

From the above figure, it is seen that the leader can lead the followers to reach the detectable range of the fixed target and simultaneously avoid the fixed obstacle. After the cluster moves to a position closer to the target, the pursuit UAVs cooperate and create a round-up formation for the target, thus completing the mission. The final distances between the pursuit UAVs and the target, as well as the success rate, are shown in Table 7.

Table 7 shows that the success rate $S_{r}$ is 82.02%, which is a bit lower than that in the scenario with no obstacle and a fixed target. This is because of the obstacle, which imposes difficulty on the round-up mission.

#### 4.4.3. Performance under the Scenario with a Fixed Target and a Moving Obstacle

In this experiment, the scenario includes a fixed target which must be tracked, as well as a moving obstacle. The goal of the cluster is to round up the fixed target while avoiding the moving obstacle. The initial position of the leader is set as $\left(100,100\right)$ m, the initial position of the target is set as (400,400) m, and the initial position of the obstacle is set as $\left(300,300\right)$ m. The velocity direction vector of the obstacle is $\left(-\frac{1}{\sqrt{2}},-\frac{1}{\sqrt{2}}\right)$, and the magnitude of the velocity of the obstacle is 1 m/s.

**Figure 21:**
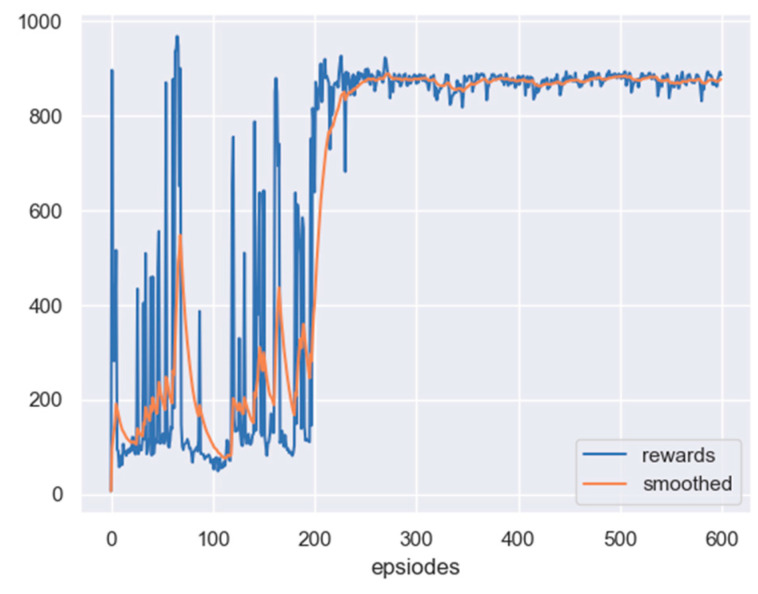
The reward curve of the leader in PPO training: a fixed target and a moving obstacle.

Obviously, the leader in this case also needs to be trained based on the PPO, and the relevant reward curve is shown in Figure 21.

**Figure 22:**
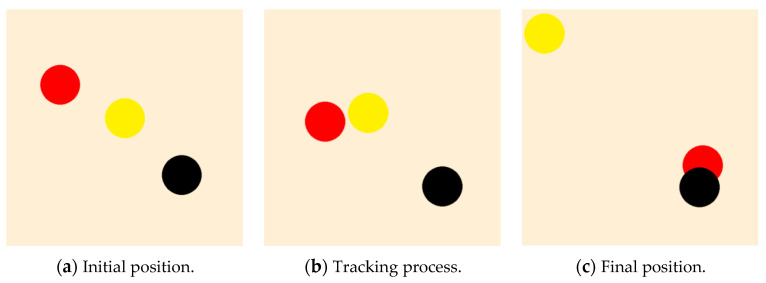
The display of the leader after PPO training: a fixed target and a moving obstacle.

From Figure 21, it can be seen that the reward curve converges after 600 times of training, and the leader is expected to reach the detectable area of the target while simultaneously avoiding the obstacle. A diagram of the leader, target, and the moving obstacle after training can be seen in Figure 22.

**Figure 23:**
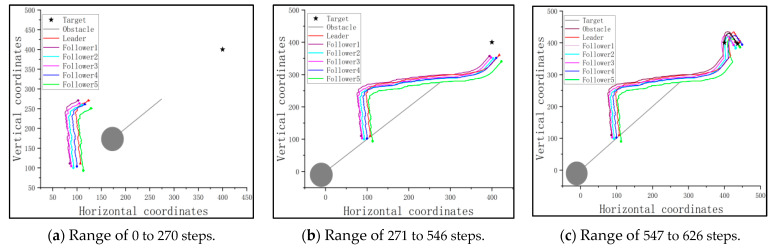
The traces of the pursuit cluster and the target with a PPO-trained leader: a fixed target and a moving obstacle.

After the introduction of the trained leader, the followers should follow the path of the leader to track the target and avoid the obstacle. Once the cluster can detect the target, the target will be rounded up, based on the consistency protocol. The corresponding traces are shown in Figure 23, where the gray range represents the impact range of the obstacle.

From Figure 23a, it can be seen that the leader leads the cluster to avoid the obstacle by turning at a specific angle. Additionally, as the obstacle and the cluster move, the cluster finds a suitable path to approach the target and avoid the obstacle, as shown in Figure 23b. Finally, with the help of the consistency protocol, the cluster rounds up the target, thus completing the mission, as illustrated in Figure 23c. The final distances between the pursuit UAVs and the target, as well as the success rate, are shown in Table 8.

Table 8 shows that the success rate $S_{r}$ is 83.35%, which is a bit higher than that in the scenario with a fixed obstacle. This is because the speeds of the pursuit UAVs are faster than that of the obstacle, and the moving trend of the obstacle also affects the result. However, due to the presence of the obstacle, the success rate here is still lower than that in the scenario with no obstacle.

## 5. Conclusions

To deal with potential failure when rounding up a target for the multi-UAV cluster, this paper proposes a consistent round-up strategy based on PPO path optimization. The involved multi-UAV cluster adopts the leader–follower structure. In regards to the condition when the target is out of the detectable range or in which an obstacle exists nearby, the leader should be trained using the PPO algorithm to guide the followers to approach the target, as well as to avoid the obstacles. Once the cluster can detect the target, the pursuit cluster can round up the target through a designed formation, based on the Apollonian circle and the consistency protocol. In the experiments, the success rates of the pursuit multi-UAV group for rounding up the target are maintained above 80% in the four testing scenarios. Additionally, we found that the moving trend and the existence of the obstacle will affect the performance of the pursuit cluster in different directions.

## Figures and Tables

**Table 1:** Setting of training hyperparameters.

Training Hyperparameters	Value
Batch Size	5
Critic Network Learning Rate	0.0003
Actor Network Learning Rate	0.0003
Discount Factor	0.95
Soft-Update Coefficient	0.01
Number of Neurons in Hidden Layer 1	256
Number of Neurons in Hidden Layer 2	256
Max Training Episode	600
Max Test Episode	20
Max Episode Length	20

**Table 2:** Setting of environmental parameters.

Experimental Parameters	Value
Acceleration of Pursuer	1
Acceleration of Target	1
Max Velocity of Pursuer	3.5
Max Velocity of Target	2.5
Velocity of Obstacle	1
Detectable Range of Pursuer	50
Detectable Range of Target	10
Impact Range of Obstacle	50
Initial Horizontal Velocity Range of Pursuer	[2, 3]
Initial Vertical Velocity Range of Pursuer	[2, 3]
Leader’s Training Environment Size	[600 × 600]

**Table 3:** Function parameters.

Experimental Parameters	Value
$k_{\alpha }$	0.4
$k_{d}$	0.2
$k_{\gamma }$	1
*n*	6

**Table 4:** The final distances and the success rate with the method of consistency protocol: no obstacle and a fixed detectable target.

$d_{l}$	$d_{f1}$	$d_{f2}$	$d_{f3}$	$d_{f4}$	$d_{f5}$	$S_{r}$
14.08	13.35	8.70	11.36	9.96	9.13	90.10%

**Table 5:** The final distances and the success rate with a PPO-trained leader: no obstacle, but an undetectable fixed target.

$d_{l}$	$d_{f1}$	$d_{f2}$	$d_{f3}$	$d_{f4}$	$d_{f5}$	$S_{r}$
11.88	13.6	13.70	11.01	12.05	10.47	82.52%

**Table 6:** The final distances and the success rate with a PPO-trained leader: no obstacle, but a moving target.

$d_{l}$	$d_{f1}$	$d_{f2}$	$d_{f3}$	$d_{f4}$	$d_{f5}$	$S_{r}$
10.03	11.76	11.99	9.53	13.73	14.09	84.35%

**Table 7:** The final distances and the success rate with a PPO-trained leader: a fixed target and a fixed obstacle.

$d_{l}$	$d_{f1}$	$d_{f2}$	$d_{f3}$	$d_{f4}$	$d_{f5}$	$S_{r}$
11.98	12.98	10.95	11.26	14.39	12.9	82.02%

**Table 8:** The final distances and the success rate with a PPO-trained leader: a fixed target and a moving obstacle.

$d_{l}$	$d_{f1}$	$d_{f2}$	$d_{f3}$	$d_{f4}$	$d_{f5}$	$S_{r}$
10.22	10.39	13.28	11.44	14.19	12.50	83.35%

**Figure A1:**
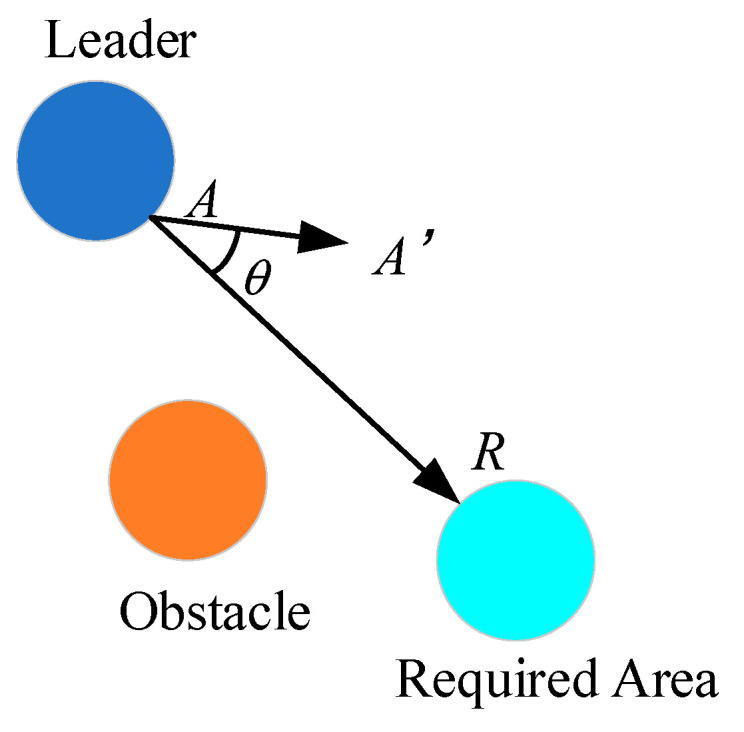
Diagram of the locations of the leader, obstacle, and the required area.

## Data Availability

The data is unavailable.

## Appendix A. Reward Design for the Leader in the PPO Algorithm

When training the leader in the cluster using PPO, the objective for the training basically contains the following issues: (a) enabling the leader to approach the detectable range of the target; (b) avoiding the obstacles; and (c) making the flight path to the target as short as possible. Therefore, the reward function in PPO can be designed as:

(A1) $$R=R_{position}+R_{direction}$$

where $\;R_{position}\;$ is the reward obtained from the leader at each position state, and $R_{direction}$ is the reward when moving in a different direction.

Since we hope that the leader can safely approach the target using the shortest path, the designed $R_{position}$ contains two components, shown as:

(A2) $$R_{position}={R_{aim}+R}_{obstacle}$$

where $R_{aim}$ reflects the time for the leader to move to the required area, and $R_{obstacle}$ guides the leader to avoid the obstacles. The reward function of $R_{aim}$ is designed as:

(A3) $$R_{aim}=\frac{1}{2}k{\left(\frac{1}{D_{a}}-\frac{1}{D_{r}}\right)}^{2}$$

where k denotes a positive constant, $D_{a}$ denotes the distance of the leader from the target, and $D_{r}$ denotes the radius of the detectable range. Moreover, for $R_{obstacle}$, it is designed as:

(A4) $$R_{obstacle}=\left\{\begin{array}{c} -\frac{1}{2}\gamma {\left(\frac{1}{D_{o}}-\frac{1}{D_{b}}\right)}^{2},\;\;\;D_{o}\leq D_{b}\\ \;\;\;\;\;\;\;\;\;\;\;\;\;\;\;\;\;\;\;0\;\;\;\;\;\;\;\;\;\;\;,\;\;\;\;D_{o}>D_{b} \end{array}\right.$$

where γ denotes a positive constant, $D_{o}$ denotes the relative distance of the leader from the obstacle, and $D_{b}$ denotes the maximum radius of the obstacle’s impact range. From the expression of $R_{obstacle}$, it can be seen that the closer the relative distance between the leader and the obstacle, the greater the penalty the obtained by the leader.

In order to bring the leader to the required area while avoiding the obstacle more quickly, the design of $R_{direction}$ should consider the best direction for the leader at each time step. Figure A1 shows the diagram of the locations of the leader, the obstacle, and the required area.

In Figure A1, it can be seen that the ideal direction for the leader is $\overset{⃑}{AR}$. If the actual direction of the leader is $\overset{⃑}{AA{}^{\prime }}$, a deviated angle θ can be obtained as follows.

(A5) $$\theta =arccos\frac{\overset{⃑}{AR}\cdot \overset{⃑}{AA{}^{\prime }}}{\left|\overset{⃑}{AR}|\cdot |\overset{⃑}{AA{}^{\prime }}\right|}$$

Thus, the design of $R_{direction}$ can be expressed as

(A6) $$R_{direction}=\frac{\frac{\pi }{2}-\theta }{180}$$

which indicates that a smaller value of θ responds to a higher reward of $R_{direction}$.

## Appendix B. Target Escape Strategy

The target which needs to be captured is set to have two moving strategies. The target is set to have a certain ability to find the pursuit UAV. Therefore, when it judges that the condition is safe, it moves along its designed flight, but when it recognizes any pursuit UAV, it will escape, and the escaping route can be expressed as:

(A7) $$\left\{\begin{array}{c} x_{t}=v_{tx}∆t+x_{td}\\ y_{t}=v_{ty}∆t+y_{td} \end{array}\right.$$

where $\left(x_{t},y_{t}\right)$ represents the target’s position,$\;(x_{td},y_{td})$ represents the position at the time when the target find itself being tracked by the pursuit UAV, and $\left(v_{tx},v_{ty}\right)$ represents the target’s velocity. The velocity $\left(v_{tx},v_{ty}\right)$ can be updated as

(A8) $$\left(v_{tx},v_{ty})=(\frac{v_{e}\sum_{i=1}^{n_{d}}v_{xi}}{\sqrt{{\left(\sum_{i=1}^{n_{d}}v_{xi}\right)}^{2}+{\left(\sum_{i=1}^{n_{d}}v_{yi}\right)}^{2}}},\frac{v_{e}\sum_{i=1}^{n_{d}}v_{yi}}{\sqrt{{\left(\sum_{i=1}^{n_{d}}v_{xi}\right)}^{2}+{\left(\sum_{i=1}^{n_{d}}v_{yi}\right)}^{2}}}\right)$$

where $n_{d}$ represents the number of the pursuit UAVs identified by the target, $\left(v_{xi},v_{yi}\right)$ represents the speed of the *i*th pursuit UAV, and $v_{e}$ represents the maximum flight speed of the target.
